# The Cerebellum in Drug-naive Children with Tourette Syndrome and Obsessive–Compulsive Disorder

**DOI:** 10.1007/s12311-021-01327-7

**Published:** 2021-09-30

**Authors:** Sankalp Tikoo, Antonio Suppa, Silvia Tommasin, Costanza Giannì, Giulia Conte, Giovanni Mirabella, Francesco Cardona, Patrizia Pantano

**Affiliations:** 1 Department of Human Neurosciences, Sapienza University of Rome, Viale dell’Università 30, 00185 Rome, Italy; 2 Department of Clinical and Experimental Sciences Section, Brescia University, Brescia, Italy; 3 IRCCS Neuromed, Pozzilli, IS Italy; 4 Department of Neuroimmunology, IRCCS Fondazione Santa Lucia, Rome, Italy

**Keywords:** Tourette syndrome, Obsessive–compulsive disorder, Cerebellum, Structural connectivity and functional connectivity, Drug-naive children

## Abstract

Tourette syndrome (TS) and obsessive–compulsive disorder (OCD) are two neurodevelopmental disorders characterized by repetitive behaviors. Our recent study in drug-naive children with TS and OCD provided evidence of cerebellar involvement in both disorders. In addition, cerebellar functional connectivity (FC) was similar in TS patients without comorbidities (TSpure) and TS patients with OCD comorbidity (TS + OCD), but differed in pure OCD patients. To investigate in detail the cerebellar involvement in the pathophysiology of TS and OCD, we explored cerebellar structural and functional abnormalities in drug-naive children with TSpure, TS + OCD, and OCD and assessed possible correlations with severity scores. We examined 53 drug-naive children, classified as TSpure (*n* = 16), TS + OCD (*n* = 14), OCD (*n* = 11), or controls (*n* = 12). All subjects underwent a multimodal 3T magnetic resonance imaging examination. Cerebellar lobular volumes and quantitative diffusion tensor imaging parameters of cerebellar peduncles were used as measures of structural integrity. The dentate nucleus was selected as a region of interest to examine cerebello-cerebral functional connectivity alterations. Structural analysis revealed that both TSpure and TS + OCD patients had higher fractional anisotropy in cerebellar peduncles than controls. Conversely, OCD patients were characterized by lower fractional anisotropy than both controls and TSpure and TS + OCD patients. Lastly, cerebellar functional connectivity analysis revealed significant alterations in the cerebello-thalamo-cortical circuit in TSpure, TS + OCD, and OCD patients. Early cerebellar structural and functional changes in drug-naive pediatric TSpure, TS + OCD, and OCD patients support a primary role of the cerebellum in the pathophysiology of these disorders.

**Supplementary Information:**

The online version contains supplementary material available at 10.1007/s12311-021-01327-7.

## Introduction

Tourette syndrome (TS) is a neuropsychiatric disorder characterized by multiple motor/phonic tics that are persistent for a year or more [1]. TS is accompanied by several comorbidities, predominantly obsessive–compulsive disorder (OCD) [2, 3]. OCD may also manifest alone as a disorder characterized by recurrent intrusive thoughts/urges and compulsions [4]. Repetitive, situation-inappropriate, and distressful movements or behaviors are the hallmark of both TS and OCD. However, despite many similarities in phenomenology and clinical course, several differences have been highlighted between TS and OCD in the literature over the last couple of decades [5]. Such differentiations include, in TS + OCD, higher presence of touching, symmetry searching and “just-right” repetitive behaviors [6, 7], compulsions less frequently preceded by obsessions and more often associated to sensory-cognitive phenomena—such as achieving a sense of completion [8] and greater neurological soft signs with poorer motor coordination and sensory integration skills [9]. In this context, clinical and neuropsychological investigations have offered limited insight as to whether TS + OCD may represent an intermediate phenotype of TS and OCD with shared pathophysiological abnormalities, a subtype of one of the two disorders or even a distinct entity [10].

To address this issue, we recently used an exploratory approach to directly compare resting-state functional connectivity (rs-FC) patterns in independent cohorts of drug-naive TS patients without comorbidities (TSpure), TS patients with OCD comorbidity (TS + OCD), and pure OCD [11]. Our previous study design minimized relevant confounding factors, such as the effect of heterogeneous disease duration and chronic drug treatment, by studying pediatric cohorts of drug-naive patients and age-matched controls. By examining functional connectivity (FC) in seven resting-state networks of interest, we reported functional changes in the cerebellar network in the above cohorts [11]. We demonstrated that all patient groups were characterized by increased cerebellar FC as compared to controls. While the rs-FC pattern was comparable in TSpure and TS + OCD groups, the OCD group had higher cerebellar FC than both the TS subgroups. Additionally, cerebellar FC changes were correlated with the clinical scales in the two disorders, i.e., negatively with tic severity and positively with compulsive scores. Overall, our findings led us to two conclusions. First, TSpure and TS + OCD patients share common patterns of rs-FC that distinguish them from OCD patients. Second, despite the pathophysiological involvement of the cortico-striato-thalamo-cortical circuit (CSTC) in TS and OCD [12–15], the cerebellum also plays an integral role, in line with previous neuroimaging studies [16–19]. Cerebellar involvement in motor, cognitive, and emotional domains has been widely acknowledged [20]. However, the precise role of the cerebellum in tics and compulsions has not been investigated in detail, thus warranting direct comparisons of cerebellar structural and functional changes between drug-naive children with TSpure, TS + OCD, and OCD.

In the present study, we therefore explored structural changes in cerebellar gray matter (GM) lobules and white matter (WM) fiber integrity of cerebellar peduncles. We also analyzed the FC of the dentate nucleus (DN) with respect to the whole brain. Lastly, we investigated the possible association between brain alterations and clinical severity. To provide insight into early pathophysiological cerebellar changes in TS and OCD, we meticulously recruited drug-naive pediatric patients and assessed cerebellar structural and functional connectivity prior to any medication or behavioral intervention.

## Methods

### Participants

We studied 70 children: 53 of them were included in this study, while 17 children were excluded due to either severe head movement (*n* = 8) or inability to complete the magnetic resonance imaging (MRI) scan (*n* = 9). The study includes 16 TSpure (age: 9.7 ± 2.1, M/F: 15/1), 14 TS + OCD (age: 10.2 ± 2.1, M/F: 10/4), 11 OCD (age: 10.7 ± 2.5, M/F: 7/4), and 12 controls (age: 10 ± 1.2, M/F: 3/9) with episodic tension headache who were headache-free during the MRI scan. Only those controls were enrolled in the study who had no current or prior history of any neurological or psychiatric disorder including tics and OCD. All subjects were enlisted from the child and adolescent neuropsychiatry outpatient clinic at the Department of Human Neurosciences, Sapienza University of Rome, Italy. Inclusion criteria were (a) drug-naivety; (b) right-handedness; and (c) normal cognitive profile (IQ ≥ 70). Exclusion criteria were (a) attention-deficit hyperactivity disorder, autism spectrum disorder, and any axis I psychiatric disorder comorbidity such as schizophrenia, schizoaffective disorder, bipolar disorder, major depression disorder, and eating disorders; (b) prior behavioral treatment; or (c) contraindications to MRI.

Cognitive evaluation for all participants was assessed by means of the Wechsler intelligence scale for children III (WISC-III) full scale. Diagnosis was made according to DSM-5 criteria [1] by a neuropsychiatrist experienced in assessing pediatric TS, OCD, and related comorbidities. Tic and OCD symptom severity was assessed using the Yale global tic severity scale (YGTSS) total tic score (TTS) (range, 0–50) (YGTSS-TTS: range 0–50, without impairment score) and the children’s Yale-Brown obsessive–compulsive scale (CYBOCS), respectively. For clinical demographics of the participants refer to Table 1. The presence of other developmental disorders including ADHD or psychiatric disorders other than OCD was ruled out by means of the K-SADS-PL parental interview administered to both parents [21]. This study was approved by the institutional review board of Sapienza University of Rome. Written informed consent was obtained from all parents/guardians in accordance with the Declaration of Helsinki.

**Table 1:** Demographic variables and clinical characteristics

Variables	TSpure (*n* = 16)	TS + OCD (*n* = 14)	OCD (*n* = 11)	Ctrls (*n* = 12)	TS pure vs Ctrls	TSpure vs OCD	TS + OCD vs Ctrls	TS + OCD vs OCD	TSpure vs TS + OCD	OCD vs Ctrls
^*^Age (years)	9.7 ± 2.1	10.2 ± 2.1	10.7 ± 2.5	10 ± 1.2	*p* = 0.26	*p* = 0.25	*p* = 0.49	*p* = 0.61	*p* = 0.44	*p* = 0.57
^**^Male/female	15/1	10/4	7/4	3/9	***p* < 0**.**001**	***p* = 0.05**	***p* = 0.02**	*p* = 0.68	*p* = 0.10	*p* = 0.06
^***^YGTSS-TTS score (0–50)	17.5 ± 6.7	18.1 ± 10.8	0.72 ± 1.6	–	–	***p* < 0.001**	–	***p* < 0.001**	*p* = 0.85	–
^***^CYBOCS score (0–40)	0.25 ± 0.7	16.4 ± 6.1	19.4 ± 7.5	–	–	***p* < 0 .001**	–	*p* = 0.53	***p* < 0.001**	–

Data are expressed as mean (*M*) ± standard deviation (*SD*)

*YGTSS-TTS* Yale global tic severity scale Total Tic Score, *CYBOCS* children’s Yale-Brown obsessive–compulsive scale, *OCD* obsessive–compulsive disorder, *TSpure* Tourette syndrome without obsessive–compulsive disorder symptoms, *TS* + *OCD* Tourette syndrome with obsessive–compulsive disorder symptoms, *Ctrls* controls (age-matched)

^*^Age differences were assessed between groups via Kruskal–Wallis and post hoc Mann–Whitney *U* test

^**^Gender differences were assessed via chi-square (χ^2^) test

^***^Mann–Whitney (*U*) test between the clinical scores of TSpure, TS + OCD, and OCD

Tic severity was evaluated by summing the motor and phonetic tics (without impairment scores) as per the YGTSS guidelines. Significant *p* values are highlighted in bold font (*p* < 0.05)

### MRI Acquisition

After clinical evaluation, all subjects underwent a 3 T MRI scan (Magnetic Verio; Siemens, Erlangen, Germany) using a standardized protocol and a 12-channel head coil designed for parallel imaging (GRAPPA, generalized autocalibrating partial parallel acquisition). Subjects were scanned in a supine head-first position with symmetrically placed cushions to minimize head motion. The MRI protocol included the following sequences: (a) high-resolution 3D, T1-weighted (3DT1) MPRAGE: repetition time (TR) = 1900 ms, echo time (TE) = 2.9 ms, flip angle = 9°, field of view (FOV) = 260 mm^2^, matrix = 256 × 256, 176 sagittal slices 1 mm thick, no gap; (b) diffusion-tensor imaging (DTI, single-shot echo-planar spin-echo sequence, with one b = 0 and 30 gradient directions, b = 0 and 1000 s/mm^2^, TR = 12,200 ms, TE = 94 ms, FOV = 192 mm, matrix = 96 × 96, 72 axial 2-mm thick slices, no gap); and (c) resting-state functional magnetic resonance imaging (rs-fMRI): TR = 3000 ms, TE = 30 ms, flip angle = 89°, 64 × 64 matrix, 50 contiguous axial slices 3 mm thick, 140 vol, acquisition time = 7 min. During the MRI scan, subjects were asked to lie down, close their eyes, and remain awake and relaxed.

### Data Analysis

Images were analyzed via FMRIB’s software library (FSL) version 6.0.1. 3DT1 images were brain extracted using the brain extraction toolbox (BET) and segmented into GM, WM, and cerebrospinal fluid (CSF). An age-specific pediatric template was created via the cerebromatic toolbox [22], with age, sex, and scanner strength as covariates.

#### Lobular Volume Analysis

Cerebellar lobular volumes were calculated using the SUIT toolbox [23]. The procedure involved cropping and isolating the cerebellum from the 3DT1 anatomical images for each subject. Each cropped image was subsequently normalized into SUIT space using generated flowfield and affine transformations. Lastly, the probabilistic cerebellar atlas was resliced back into the individual subject space, resulting in GM measurements of 13 bilateral regions of the cerebellum (lobules I–IV, V, VI, crus I, II, VIIb, VIIIa, VIIIb, IX, X, dentate, interposed nucleus, and fastigial nucleus) and 8 vermis regions. Values of the extracted cerebellar regions were normalized to the total intracranial volume to reduce head size variability.

#### WM Analysis

Tract-based spatial statistics (TBSS) [24] was employed to evaluate WM integrity. Head movement- and eddy-corrected images were fitted to the tensor model at each voxel to calculate fractional anisotropy (FA) and mean diffusivity (MD) maps using DTIFIT. Since our subjects were children, the FA target image was unsuitable. Hence, the most representative target image (i.e., the one that required the least amount of warping to match every other subject) across the four groups was identified and all subject-specific FA and MD maps were non-linearly aligned to this target image. Lastly, a WM skeleton was generated from the mean FA image by thresholding at 0.2 to exclude GM or CSF. Automatic tract-specific quantification using the John Hopkin’s University (JHU) WM tractography atlas was performed to identify the inferior cerebellar peduncles (ICP), middle cerebellar peduncles (MCP), and superior cerebellar peduncles (SCP), which were used as masks to restrict DTI analysis. Mean FA and MD values were then computed.

#### Functional MRI Analysis

The functional MRI expert analysis tool (FEAT) was used to preprocess rs-fMRI data. After discarding the first 3 volumes, rs-fMRI data were slice-time corrected, motion parameters estimated via MCFLIRT, spatially smoothed (Gaussian kernel of full-width half maximum = 8 mm), and high pass temporal filtered (100.0 s). Next, the rs-fMRI data were subjected to ICA-based noise reduction strategy called ICA-AROMA (automatic removal of motion artifacts) [25]. This method uses an automatic classifier that categorizes each component as either BOLD signal or artifact, based on its high frequency content > 35%, correlation with realignment parameters (RP) derived from MCFLIRT, edge and CSF fractions. Additionally, the mean time courses of WM and CSF were calculated from the rs-fMRI images and regressed out via fsl_glm. The rs-fMRI data were then subjected to a bandpass filter at (0.01–0.09) Hz and finally normalized onto the customized T1 template space.

For seed-based analysis, a bilateral spherical seed of the DN (4-mm radius) was created using the coordinates of the left and right dentate (− 18, − 58, − 34 and 18, − 56, − 34, respectively) in MNI space, consistent with our previous work [26, 27]. The dentate seed was then affine-transformed to the native space of each subject and its anatomical location was carefully checked on functional images. The mean time series of the bilateral dentate seed were computed for each subject and inserted into a general linear model (GLM) to generate seed-based correlation maps.

### Statistical Analysis

The Kruskal–Wallis test and post hoc Mann–Whitney *U* test were performed to assess between-group differences with respect to age, followed by the chi-square test to check for inter-group differences with respect to sex. Differences in clinical scores between groups were analyzed via Mann–Whitney *U* test. Statistical Package for the Social Sciences (SPSS-25.0) was used to compute statistical analyses. Results were Bonferroni corrected at *p* < 0.05.

#### Lobular Volume Analysis

Differences in normalized cerebellar GM volumes between study cohorts were calculated using MANCOVA followed by a post hoc two-sample *t*-test. Lastly, Pearson’s correlation was computed between the cerebellar lobules that significantly differed from controls and clinical scores. In all analyses, age and gender were included as covariates. Results were Bonferroni corrected at *p* < 0.05.

#### WM Analysis

To compute between-group differences in FA and MD in the three cerebellar peduncles, a GLM was constructed with age and gender as covariates. An ANOVA followed by a two-sample *t*-test was run to investigate inter-group differences via a non-parametric approach (applying, 5000 permutations) using Randomise tool [28] of FSL. Results were false discovery rate (FDR)-corrected with a *p* value < 0.05. For the correlation analysis, the mean values of significantly altered FA and MD between patients and controls were extracted. Pearson’s correlation was computed between mean FA and MD values and severity scores, and results were Bonferroni corrected at *p* < 0.05.

#### Dentate Nucleus Functional Connectivity (DNFC)

To evaluate inter-group differences in terms of DNFC, ANOVA was performed non-parametrically via Randomise tool [28] (*n* = 5000) using a GLM with age and sex as covariates to map brain areas that significantly differed between groups. The ANOVA-derived map was binarized to build a mask, at *p* < 0.05 after FDR correction. Furthermore, two-sample *t*-tests were computed using the same non-parametric approach to evaluate between-group differences within this mask. Differences were considered significant with a *p* value < 0.05 after FDR correction. The mean *z* scores of the significantly altered DNFC between patients and controls were extracted for each subject and Pearson’s correlation was computed with the severity scale. Results were Bonferroni corrected at *p* < 0.05.

## Results

TSpure, TS + OCD, and OCD patients and controls did not statistically differ in terms of age (*H* (3) = 2.1, *p* = 0.54). Conversely, sex distribution was uneven between TSpure and controls (χ^2^ [1, *N* = 28] = 14.1, *p* < 0.001), TS + OCD and controls (χ^2^ [1, *N* = 26] = 5.5, *p* = 0.02), and TSpure and OCD patients (χ^2^ [1, *N* = 27] = 3.9, *p* = 0.05). The Mann–Whitney *U* test revealed that YGTSS-TTS scores between TSpure and TS + OCD were comparable (*U* = 108.5, *p* = 0.88). Similarly, CYBOCS scores were comparable between OCD and TS + OCD patients (*U* = 65, *p* = 0.53) (Table 1). According to the CYBOCS checklist, obsessive symptomatology was similar in OCD and TS + OCD patients, except for hoarding/saving obsessions and miscellaneous obsessions (e.g., fear of saying or not saying something), which were respectively present in 40% (4/10) and 50% (5/10) of the OCD group as compared to the 14.3% (2/14) of the TS + OCD group. The most frequently observed types of compulsions in patients with TS + OCD were checking rituals and symmetry searching in 85.7% (12/14), followed by repeating rituals in 78.6 (11/14), touching and “just right phenomena” in 71.4% (10/14), and washing compulsions in 57.1% (8/14). In the OCD group, compulsion patterns were similarly represented with the notable exception of washing rituals, which were identified in 80% (8/10) and of touching behaviors which were less common (50%).

### Patients vs Controls

#### Lobular Volume Analysis

**Fig. 1:**
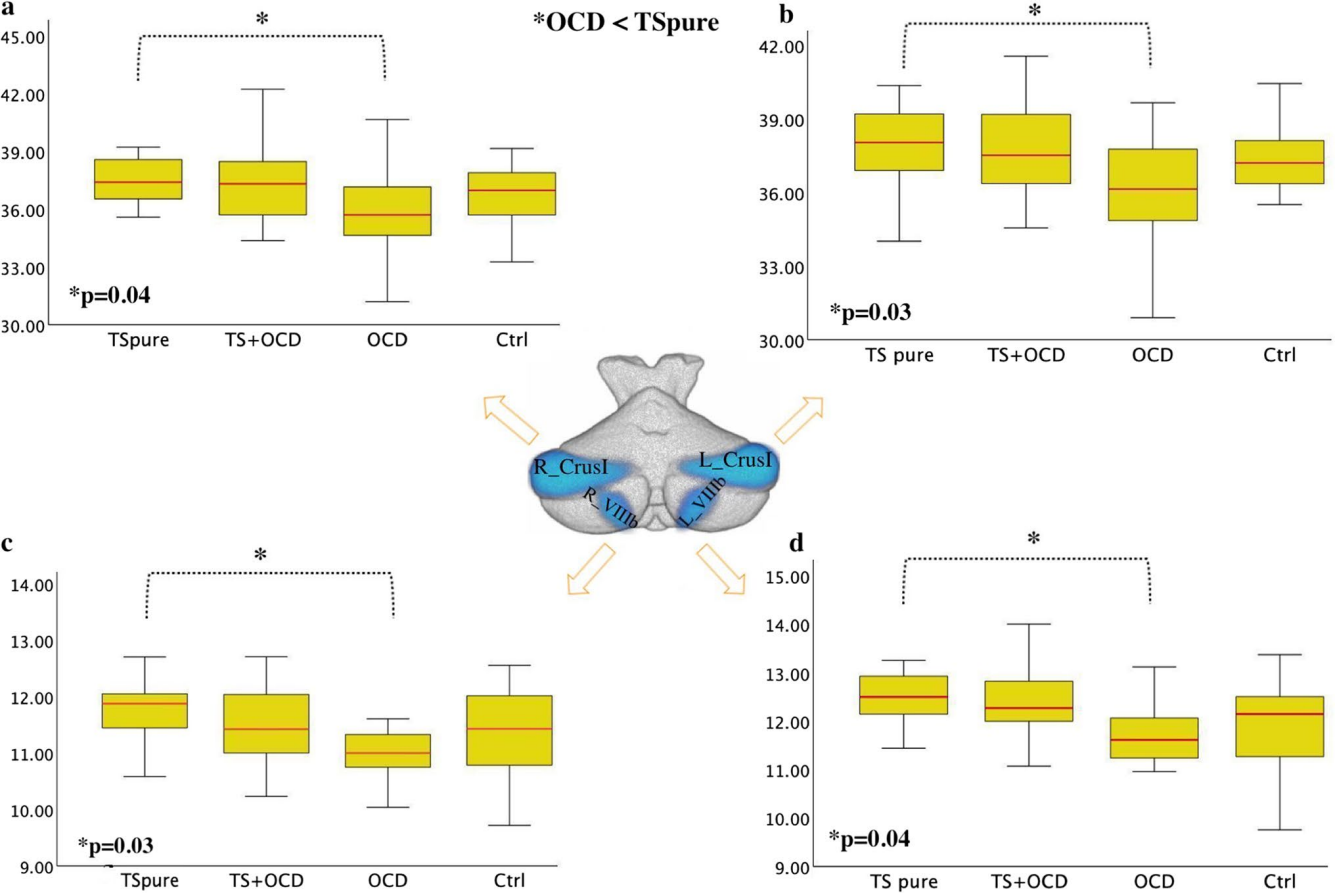
**Cerebellar lobular analysis**: Boxplots depicts the cerebellar volumetric differences between TSpure, TS + OCD, OCD, and Ctrls. Gray matter values of each cerebellar lobule were extracted via SUIT cerebellar atlas and assessed for inter-group differences. OCD patients had decreased cerebellar gray matter volume than TSpure as indicated by the blue color at **a)** right crus I, **b)** left crus I, **c)** right VIIIb, and **d)** left VIIIb. The median marks the mid-point of the data and is shown by the red line. The upper and lower whiskers represent the highest and the lowest cerebellar gray matter values, respectively. Significance was set at *p* < 0.05, Bonferroni corrected

No significant differences were found in either global or regional cerebellar volumes between patients and controls (Fig. 1, Table 2).

**Table 2:** Cerebellar gray matter changes between OCD and TSpure patients

Cerebellar volume (mL)	TSpure^*^ (355.4 ± 11.3)		TS + OCD (354.7 ± 11.3)	OCD^*^ (341.8 ± 21.2)	Ctrls (345.7 ± 19.4)	*F*/*p* value 2.4/0.08 ^*^OCD < TSpure
R_Crus I	37.51 ± 1.2	37.5 ± 1.8		35.9 ± 2.5	36.9 ± 2.1	2.9/0.04^*^
L_Crus I	37.9 ± 1.6	37.7 ± 2.1		36.1 ± 2.5	37.2 ± 1.6	3.1/0.03^*^
R_lobule VIIIb	12.0 ± 0.6	11.5 ± 0.7		11.1 ± 0.7	11.4 ± 0.8	3.3/0.04^*^
L_lobule VIIIb	12.5 ± 0.5	12.4 ± 0.9		11.7 ± 0.6	12.0 ± 0.9	2.7/0.02^*^

The above table depicts significant cerebellar gray matter (GM) differences in bilateral crus I and bilateral VIIIb lobule between obsessive–compulsive disorder (OCD) and TS patients without comorbidity (TSpure)

^*^Denotes significant group differences obtained via multivariate analysis of covariance (MANCOVA) followed by post hoc two-sample *t*-test. Results are Bonferroni corrected (*p* < 0.05) for multiple comparisons

#### WM Analysis

**Fig. 2:**
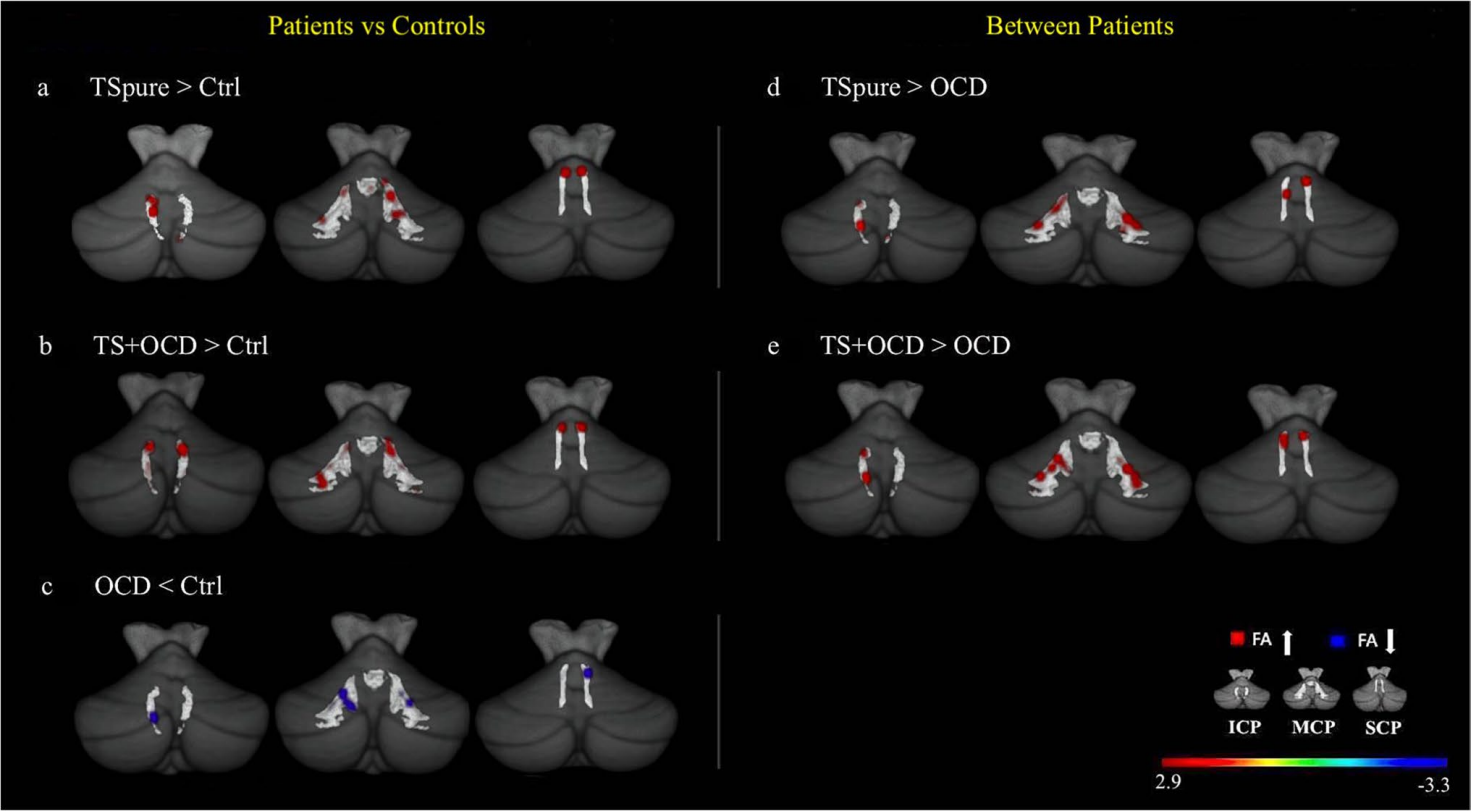
**Cerebellar peduncles white matter analysis**: Figure depicts fractional anisotropy (FA) alterations between patients and controls (**a**, **b**, **c**) and between patient cohorts (**d**, **e**) within three cerebellar tracts: inferior cerebellar peduncle (ICP), middle cerebellar peduncle (MCP), and superior cerebellar peduncle (SCP). The cerebellar peduncles are represented by light-gray color overlayed on a 3-dimensional suit template post tbss_fill for better illustration. **a)** and **b)** Red areas represent higher FA in TSpure and TS + OCD patients than controls; **c)** blue areas represent lower FA in OCD patients compared to controls; **d)** and **e)** red areas represent higher FA in TSpure and TS + OCD than OCD patients. Results were *p* < 0.05, false discovery rate (FDR) corrected. The color bar shows *t* values

When compared to controls, both TSpure and TS + OCD showed significantly higher FA (Fig. 2), whereas OCD patients exhibited lower FA in all three cerebellar peduncles (Fig. 2). MD analysis showed a similar pattern, i.e., lower values in both TSpure and TS + OCD and higher in OCD patients than controls in all cerebellar peduncles (Supplementary Fig S1). Thus, further analysis was restricted to FA changes alone.

#### Dentate Nucleus Functional Connectivity

**Fig. 3:**
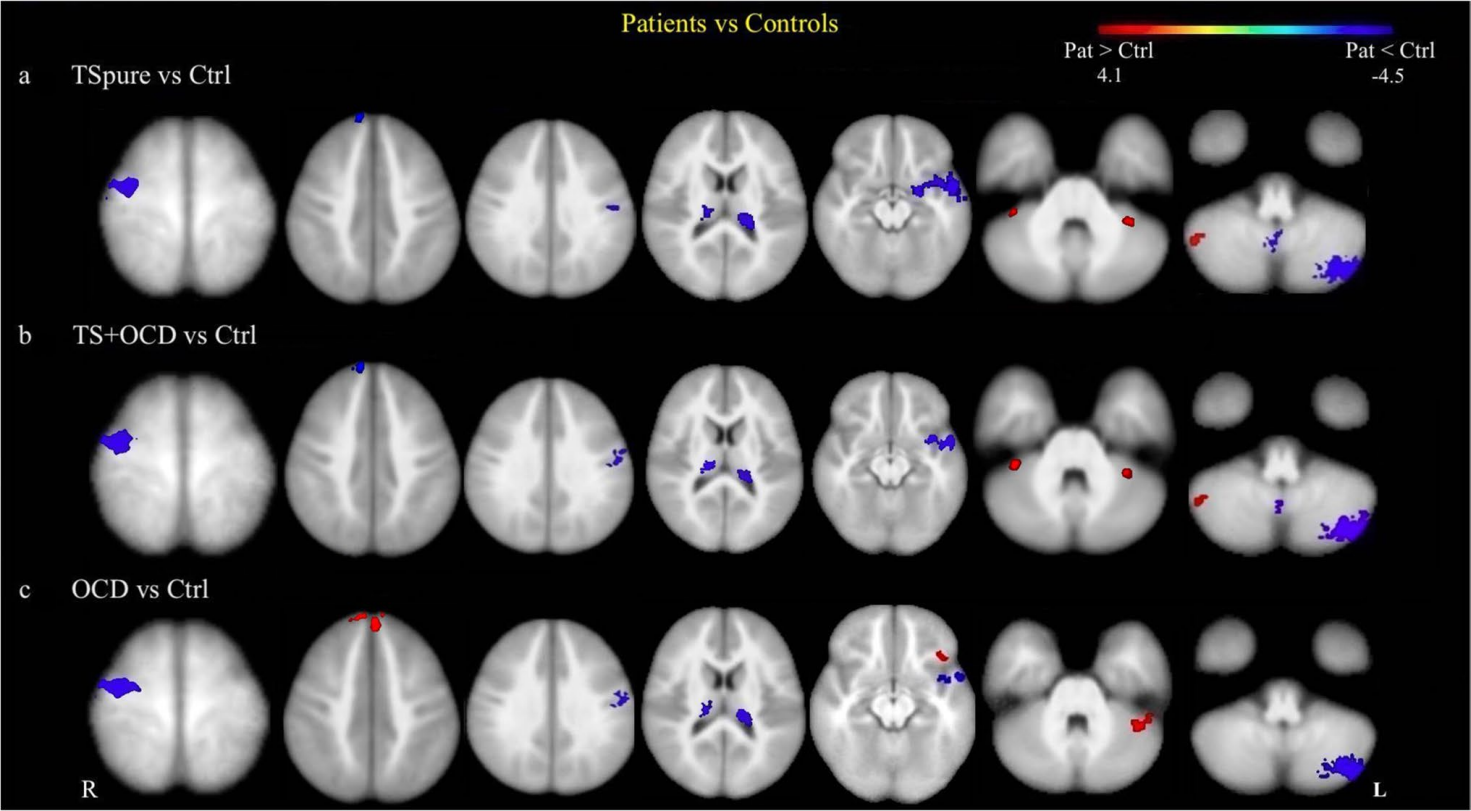
**Cerebellar functional connectivity differences between patients and controls**: Figure depicts group differences in dentate nucleus functional connectivity (DNFC) between patients and controls. Light blue color indicates areas of reduced DNFC while red indicates areas of increased DNFC, superimposed on a customized T1 template. **a)** and **b)** represents decreased DNFC in TSpure and TS + OCD patients with the right precentral gyrus, right prefrontal, left postcentral gyrus, bilateral thalamus, left inferior temporal gyrus, left lobule IX, and left crus II, and an increased DNFC with the bilateral lobule VI and right crus I compared to controls; **c)** represents decreased DNFC in OCD patients with right pre and left postcentral gyrus, bilateral thalamus, left inferior temporal gyrus, and left crus II and increased DNFC with bilateral prefrontal, left fronto-orbital cortex, and left crus I compared to Ctrls. Results were *p* < 0.05, FDR corrected. The color bar represents *t* values

As compared to controls, both TSpure and TS + OCD patients exhibited decreased DNFC with the right precentral gyrus, right prefrontal cortex (PFC), left postcentral gyrus, left inferior temporal gyrus, bilateral thalamus, left cerebellar lobule IX, and left crus II. They also showed increased DNFC with bilateral lobule VI and right crus I (Fig. 3). OCD patients, in comparison to controls, exhibited decreased DNFC with the right precentral gyrus, left postcentral gyrus, left inferior temporal gyrus, bilateral thalamus, and left crus II, a pattern similar to that observed in the TSpure and TS + OCD groups. However, in contrast to the TSpure/TS + OCD groups, OCD patients showed increased DNFC with the PFC bilaterally, left orbitofrontal cortex, and left crus I (Fig. 3). The details of voxel coordinates and significant *p* and *t* values are listed in Supplementary Table S1.

### Between-Group Comparison

#### Lobular Analysis

Compared to TSpure, OCD patients had significantly lower GM volume in crus I and VIIIb bilaterally (Fig. 1, Table 2).

#### WM Analysis

TSpure and TS + OCD patients did not show significant differences in FA in any of the cerebellar peduncles. In contrast, both TSpure and TS + OCD exhibited higher FA than OCD patients in all three cerebellar peduncles (Fig. 2). Similarly, no MD differences were found between TSpure and TS + OCD, and both groups exhibited lower MD than OCD patients (Supplementary Fig S1).

#### Dentate Nucleus Functional Connectivity

**Fig. 4:**
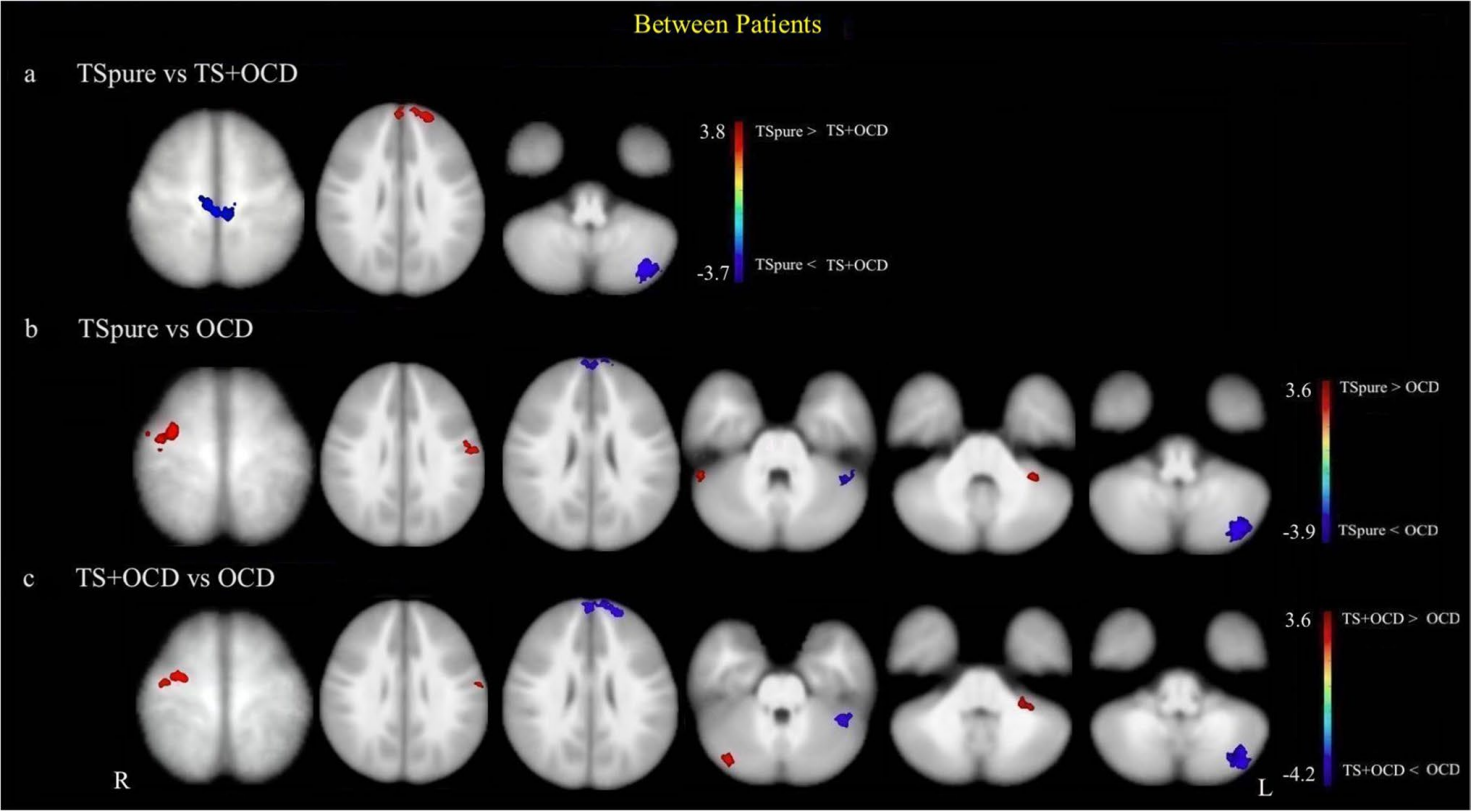
**Cerebellar functional connectivity differences between patient groups**: Figure depicts group differences in seed-based dentate nucleus functional connectivity (DNFC) between patient cohorts. Light blue color indicates areas of reduced DNFC while red indicates areas of increased DNFC, superimposed on a customized T1 template. **a)** represents decreased DNFC in TSpure patients with bilateral pre-central gyrus and left crus II and an increased DNFC with bilateral prefrontal cortex compared to TS + OCD patients; **b)** and** c)** depict increased DNFC in TS pure and TS + OCD with right pre and left postcentral gyrus, left lobule VI, and right crus I and decreased DNFC with bilateral prefrontal cortex, left crus I, and left crus II in comparison to OCD patients. Results were *p* < 0.05, FDR corrected. The color bar represents *t* values

As compared to TS + OCD, TSpure patients exhibited lower DNFC with the bilateral precentral gyrus and left crus II, as well as higher DNFC with the PFC bilaterally (Fig. 4). With respect to OCD, both TSpure and TS + OCD patients exhibited higher DNFC with the right pre and left postcentral gyrus, right crus I, and left lobule VI. They also showed lower DNFC with the bilateral PFC, left crus I, and crus II (Fig. 4). The details of voxel coordinates and significant *p* and *t* values are listed in Supplementary Table S1.

### Correlations

**Fig. 5:**
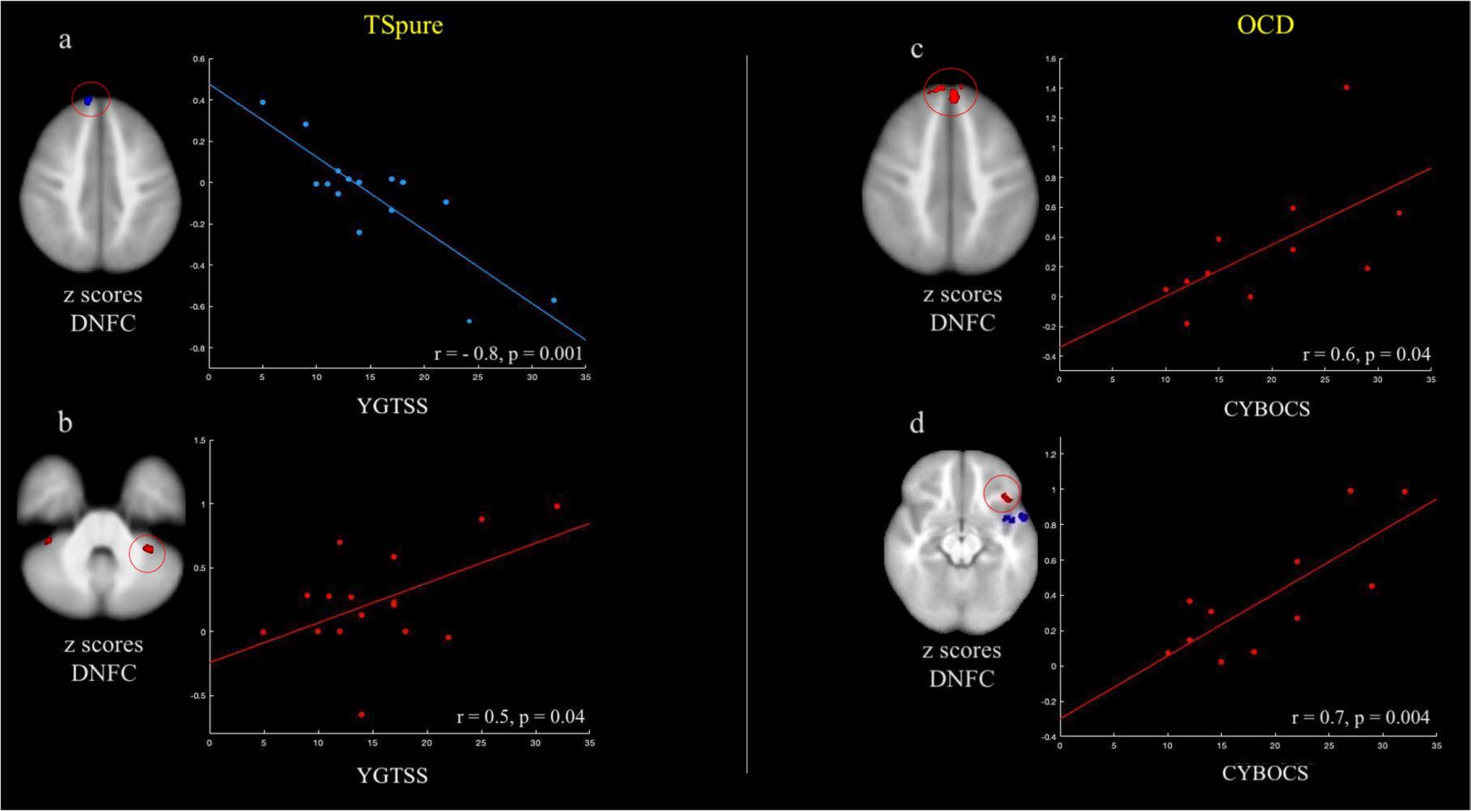
**Correlation of altered dentate nucleus functional connectivity (DNFC) with clinical measures**: Figure depicts correlation of altered DNFC (*z* scores) with clinical severity scale (Yale global tic severity scale total tic score (YGTSS-TTS) and children’s Yale-Brown obsessive–compulsive scale (CYBOCS)) in TSpure and OCD patients, respectively. Circles indicate areas of increased (red) or decreased (blue) DNFC in patients with respect to controls, whose *z* scores were correlated with clinical scores. **a)** DNFC with the right prefrontal cortex negatively correlated with YGTSS-TTS; **b** DNFC with the left lobule VI positively correlated with the YGTSS-TTS; **c)** DNFC with the bilateral prefrontal cortex positively correlated with the CYBOCS; **d)** DNFC with the left orbitofrontal cortex positively correlated with the CYBOCS. Results were *p* < 0.05, Bonferroni corrected

Neither altered cerebellar GM lobules nor altered FA significantly correlated with clinical scores in any patient cohort. In TSpure patients, YGTSS-TTS score negatively correlated with DNFC with the right PFC and positively correlated with DNFC with left lobule VI (Fig. 5). No significant correlations were found between altered DNFC in TS + OCD patients and any of the severity scores. In OCD patients, CYBOCS score positively correlated with DNFC with the bilateral PFC and left orbitofrontal cortex (Fig. 5).

## Discussion

Our study provides evidence of early-stage alterations in cerebellar structural and functional connections in drug-naive children with TSpure, TS + OCD, and OCD. We found alterations in cerebellar WM connections and FC in all patient cohorts as compared to controls. Our findings point to cerebellar involvement in the pathophysiology of TS and OCD.

### Cerebellar Lobular Volume

We did not find any significant differences in cerebellar GM volume between patients and controls. Our findings of normal cerebellar GM volume in drug-naive TS children are consistent with previous studies demonstrating no GM loss in these children [29–31]. Some studies have shown reduced cerebellar GM in TS patients, although these studies included patients with mixed comorbidity/medication profiles [16] or both children and adults [17]. The cerebellar GM loss so far reported in TS patients may reflect adaptive changes in adult brains or be a consequence of prolong exposure to drugs.

Consistent with our results, the majority of prior volumetric studies in OCD patients reported no GM changes in the cerebellum [32–34], though a few studies reported either decreased [35] or increased [36] cerebellar GM volume in OCD, which might be due to differences in methodology or patient selection.

Between-group comparison revealed lower GM in bilateral crus I and lobule VIIIb in OCD patients than in TSpure patients, indicating subtle differences in cerebellar GM between these two conditions, symmetrically affecting specific lobules, i.e., crus I and lobule VIII, which are known to contribute to cognitive and sensorimotor functions, respectively [37].

### Cerebellar WM Tracts

Both TSpure and TS + OCD patients had higher FA than controls in all three cerebellar peduncles, consistent with previous findings [38]. Conversely, OCD patients had lower FA than controls in all three cerebellar peduncles, in line with a recent study [39]. Consequently, both TSpure and TS + OCD significantly differed from OCD patients in terms of WM microarchitecture of cerebellar connections.

Considering FA as a measure of axonal package density and overall WM integrity [40], our findings suggest that axonal density increases in both TSpure and TS + OCD, whereas it decreases in OCD patients. The correlation between WM changes and symptom expression in TS and OCD is not fully understood. However, increased FA has been reported in TS in sensorimotor [38] and prefrontal regions [41] and has been associated with reduced tic severity and better motor behavior control. A mechanism of adaptive brain reorganization involving cerebellar WM connections likely aiming to control tics may thus be postulated. Conversely, the lower FA values in OCD children might represent a pathophysiological mechanism that may be partly reversed by cognitive-behavioral therapy [39]. Overall, the pediatric and drug-naive features of our cohort enabled us to detect early-stage WM abnormalities in the cerebellar peduncles of TS and OCD patients, further supporting the role of abnormal WM in the pathophysiology of these two disorders.

### Dentate Nucleus Functional Connectivity

As compared to controls, TSpure, TS + OCD, and OCD patients showed decreased DNFC with the thalamus, pre and postcentral gyri, and inferior temporal gyrus. These findings are consistent with prior research [42–46]. In TS, the sensorimotor cortex and thalamus are involved in the premonitory sensation of urges while the temporal gyrus is responsible for directing control of urge inhibition [47–49]. Our findings favor the hypothesis that decreased DNFC underpins poor control over premonitory urges and tics in TS patients. Similarly, reduced cerebellar FC with the thalamus and sensorimotor areas may underlie difficulties in controlling repetitive thoughts and actions in OCD patients. Also, prior studies have associated the decreased activity of inferior temporal gyrus with low insight level in OCD patients [50]. Thus, our findings of reduced cerebellar-temporal FC might reflect poor insight level or a lack of self-awareness in OCD patients.

With respect to controls, TSpure and TS + OCD patients had reduced cerebellar-prefrontal connectivity, whereas OCD patients had increased cerebellar-prefrontal and orbitofrontal connectivity. Consistent with our results, some prior studies have demonstrated decreased cerebellar-frontal connectivity in TS patients [51] and increased prefrontal/orbitofrontal excitability in OCD [52, 53]. In our sample, cerebellar-prefrontal connectivity negatively correlated with tic severity, whereas in OCD cerebellar-prefrontal and orbitofrontal connectivity positively correlated with compulsive scores. Thus, altered FC between the cerebellum and frontal cortices in both TS and OCD likely represents a pathophysiological correlate suggestive of widespread involvement of networks responsible for guiding goal-oriented behaviors [54]. The PFC is crucial in the ability to exert adaptive control over behavior, and hyperactivity in the frontal region likely indicates abnormal performance monitoring in OCD [55, 56]. In contrast, altered FC in the frontal cortex might explain the defective control over volitional tic behavior observed in TS [57].

When examining DNFC with other cerebellar regions, we observed lower FC with the left lobule IX and crus II and higher FC with lobule VI and crus I in TSpure, TS + OCD, and OCD patients as compared to controls, in line with prior findings demonstrating decreased intra-cerebellar FC in adult TS patients [51] and increased cerebellar FC in pediatric TS and OCD patients [11]. Additionally, we also found a positive association between increased DNFC in TSpure patients and tic severity. Cerebellar involvement in language processing and visuospatial functions is well acknowledged [37]. Our findings of increased and decreased intra-cerebellar FC may therefore indicate wide-ranging cognitive deficits in both TS and OCD patients.

Between-group comparison revealed that TSpure patients had higher DNFC with the PFC and lower DNFC with precentral and crus II regions as compared to TS + OCD patients. We speculate that DNFC differences between TSpure and TS + OCD patients could reflect a different burden of disease when associated with psychiatric comorbidity. Additionally, both TSpure and TS + OCD patients had lower cerebellar FC with the PFC but higher DNFC with sensorimotor areas as compared to OCD patients.

Overall, our findings demonstrating altered DNFC with the thalamus, various cerebellar hemisphere regions, and cerebral cortex point to a functional disconnection of the cerebello-thalamo-cortical (CTC) circuit that affects both TS and OCD patients.

### The Cerebellum in TS and OCD

Recent studies have proposed a salient role of the cerebellum in the pathogenesis of tics and compulsions [16, 18, 39]. However, the cerebellum is still underrepresented in TS and OCD research. The present study highlights abnormalities in both structural and functional cerebellar connections in TSpure, TS + OCD, and OCD patients. We demonstrated significant alterations in cerebellar microstructural WM fibers in both TS and OCD patients, with TS having higher cerebellar structural integrity than OCD patients. Furthermore, we found that cerebello-prefrontal FC is reduced in TS and increased in OCD, which suggests that the cerebellum engages with the PFC in a distinct manner in TS as compared to OCD patients. The negative vs positive correlation between cerebellar-prefrontal FC and clinical severity found in TS and OCD patients respectively further supports a different contribution of the disruption of this circuit, which is mainly involved in executive functions, to the pathophysiology of these two disorders. Lastly, our study reported no differences in cerebellar structural architecture in both the TS subcohorts. Moreover, we also found that TSpure and TS + OCD share common DNFC pattern when compared to both controls and OCD patients. Our findings suggest common pathophysiological underpinnings of the two clinical subgroups and support the hypothesis of TS + OCD as a subtype and alternative phenotype of TS.

### Limitations

First, our cohorts were characterized by an uneven gender distribution that might have influenced the results, though sex was included in the analyses as a covariate. However, our primary focus was the recruitment of drug-naive children. Second, although we instructed our TS patients to tic freely, we did not monitor the number of tics during MRI acquisition. Third, although our study consisted of 53 subjects, the number of patients per subgroup is relatively small. Lastly, since this was not a longitudinal study, we were not able to monitor structural and functional changes over the disease course. However, our study of a well-categorized drug-naive patient population represents an initial step towards better elucidating the neural basis of TS and OCD.

### Conclusions

This study shows that TS and OCD patients share a common pattern of reduced DNFC with the thalamus and cerebellar and cerebral cortices, supporting a functional disconnection of the CTC circuit in both disorders. By identifying differences in cerebellar-prefrontal FC and cerebellar peduncle microarchitecture between drug-naive pediatric TS and OCD patients, the present multimodal MRI study provides novel insights into the pathophysiological cerebellar involvement that characterizes the early phases of these two disorders.

## Supplementary Information

Below is the link to the electronic supplementary material.

Supplementary file1 (DOC 7224 KB)
