# Supplementary material for: The Cerebellum in Drug-naive Children with Tourette Syndrome and Obsessive–Compulsive Disorder
Source: Cerebellum. 2021 Sep 30;21(6):867–78. doi: 10.1007/s12311-021-01327-7 (PMC9596574; doi:10.1007/s12311-021-01327-7)
Supplement: Supplementary file 1 — Supplementary file1 (DOC 7224 KB) [file 12311_2021_1327_MOESM1_ESM.doc]

**Table S1: Altered dentate nucleus functional connectivity (DN-FC) between all groups.**

| **Anatomical**  **Region**  **F/(p-value)** | **TSpure vs Ctrls**  Contrast  Voxel coord  p value  T value | **TS+OCD vs Ctrls**  Contrast  Voxel coord  p value  T value | **OCD vs Ctrls**  Contrast  Voxel coord  p value  T value | **TSpure vs TS+OCD**  Contrast  Voxel coord  p value  T value | **TSpure vs OCD**  Contrast  Voxel coord  p value  T value | **TS+OCD vs OCD**  Contrast  Voxel coord  p value  T value |
| --- | --- | --- | --- | --- | --- | --- |
| **R- Precentral gyrus**  1.0/<0.001 | **TSpure < Ctrl**  37 76 94  0.001  -3.8 | **TS+OCD < Ctrls**  36 76 94  < 0.001  -3.7 | **OCD < Ctrls**  37 76 92  < 0.001  -3.9 | **-** | **TSpure > OCD**  43 71 91  0.001  3.6 | **TS+OCD > OCD**  41 75 94  < 0.001  3.6 |
| **Prefrontal cortex**  0.97/<0.001 | **TSpure < Ctrls**  52 121 77  0.001  -3.8 | **TS+OCD < Ctrls**  52 121 77  < 0.001  -3.6 | **OCD > Ctrls**  55 129 63  < 0.001  3.4 | **TSpure>TS+OCD**  77 124 67  < 0.001  3.9 | **TSpure < OCD**  55 129 65  < 0.001  -3.9 | **TS+OCD < OCD**  55 129 61  < 0.001  -3.5 |
| **L- Postcentral gyrus**  0.98/<0.001 | **TSpure< Ctrls**  104 77 61  0.002  -2.8 | **TS+OCD < Ctrls**  103 83 74  0.001  -3.5 | **OCD < Ctrls**  102 80 76  < 0.001  -4.3 | - | **TSpure > OCD**  98 82 81  0.001  3.6 | **TS+OCD > OCD**  102 78 62  0.005  2.8 |
| **Bi Precentral gyrus**  0.97/<0.001 | **-** | **-** | **-** | **TSpure<TS+OCD**  63 67 97  0.001  -3.6 | **-** | **-** |
| **L- Thalamus**  0.96/<0.001 | **TSpure< Ctrls**  70 61 55  0.001  -3.6 | **TS+OCD < Ctrls**  71 65 54  0.001  -3.7 | **OCD < Ctrls**  72 63 57  < 0.001  -4.0 | **-** | **-** | **-** |
| **R- Thalamus**  0.98/<0.001 | **TSpure < Ctrls**  53 69 57  0.003  -2.9 | **TS+OCD < Ctrls**  51 67 55  0.001  -3.3 | **OCD < Ctrls**  49 68 59  < 0.001  -3.5 | **-** | **-** | **-** |
| **L- Inferior temporal gyrus**  0.97/<0.001 | **TSpure < Ctrls**  99 86 33  0.001  -3.5 | **TS+OCD < Ctrls**  88 88 35  < 0.001  -4.3 | **OCD < Ctrls**  96 89 41  < 0.001  -3.8 | **-** | **-** | **-** |
| **L- Orbitofrontal cortex**  0.97/<0.001 | - | - | **OCD > Ctrls**  90 97 34  < 0.001  4.1 | **-** | **-** | **-** |
| **R- lobule VI**  0.98/0.001 | **TSpure > Ctrls**  50 47 30  < 0.001  3.5 | **TS+OCD > Ctrls**  33 59 25  < 0.001  3.8 | **-** | **-** | **-** | **-** |
| **L- lobule VI**  0.97/<0.006 | **TSpure > Ctrls**  82 55 25  0.001  3.2 | **TS+OCD > Ctrls**  77 57 19  0.001  3.4 | **-** | **-** | **TSpure > OCD**  82 55 23  0.003  2.8 | **TS+OCD > OCD**  82 55 21  < 0.001  3.0 |
| **R- Crus I**  0.98/<0.001 | **TSpure > Ctrls**  25 52 25  0.001  3.4 | **TS+OCD > Ctrls**  33 31 29  < 0.001  3.9 | **-** | **-** | **TSpure > OCD**  24 56 27  0.002  3.0 | **TS+OCD > OCD**  33 21 28  < 0.001  3.2 |
| **L-Crus I**  0.98/<0.001 | **-** | **-** | **OCD > Ctrls**  87 51 27  0.001  -3.6 | **-** | **TSpure < OCD**  92 51 28  < 0.001  -3.8 | **TS+OCD < OCD**  89 51 26  < 0.001  -4.2 |
| **L-IX**  0.97/<0.001 | **TSpure < Ctrls**  60 46 16  0.001  -3.6 | **TS+OCD < Ctrls**  59 45 18  0.003  -2.7 | **-** | **-** | **-** | **-** |
| **L-CrusII**  0.98/<0.001 | **TSpure < Ctrls**  85 35 11  < 0.001  -4.2 | **TS+OCD < Ctrls**  89 32 13  < 0.001  -4.5 | **OCD < Ctrls**  85 36 10  0.001  -3.6 | **TSpure<TS+OCD**  74 42 19  0.001  -3.8 | **TSpure < OCD**  88 34 11  < 0.001  -3.6 | **TS+OCD < OCD**  92 38 13  <0.001  -3.1 |

The above table depicts brain regions with abnormal DN-FC between all 4 groups: TSpure: Tourette syndrome patients without comorbidity, TS+OCD: Tourette syndrome patients with Obsessive compulsive disorder comorbidity, OCD: pure Obsessive compulsive disorder patients and Ctrls: age-matched controls; (FDR corrected for multiple comparisons, p < 0.05).

**Fig S1**


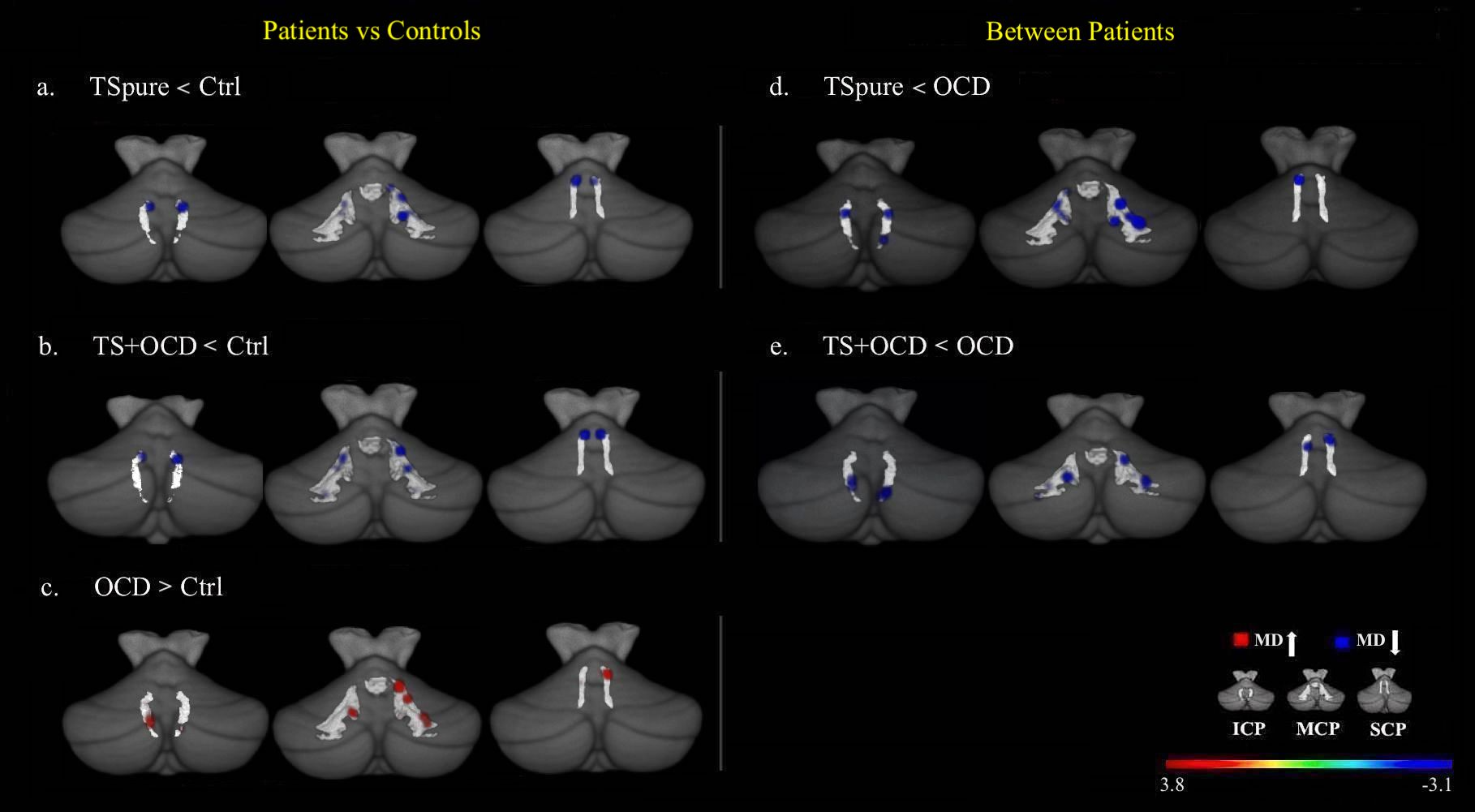


Mean diffusivity (MD) alterations between patients and controls (a, b, c) and between patient cohorts (d, e) within three cerebellar tracts: inferior cerebellar peduncle (ICP), middle cerebellar peduncle (MCP) and superior cerebellar peduncle (SCP). The cerebellar peduncles are represented by light-grey color overlayed on a 3-dimensional suit template post tbss_fill for better illustration.

**a** and **b)** blue areas represent lower MD in TSpure and TS+OCD patients than controls; **c)** red areas represent higher MD in OCD patients compared to controls; **d** and **e)** blue areas represent lower MD in TSpure and TS+OCD than OCD patients. Results were p < 0.05, false discovery rate (FDR) corrected. The color-bar shows t values.
